# Compound Dislocation of the Long Bones Considered with Especial Reference to the Value of Resections

**Published:** 1857-12

**Authors:** 


*Compound Dislocation of the Long Bones considered with especial reference to the value of Resections.* By Frank Hastings Hamilton, M. D., Prof. of Surgery in the Med. Department of the University of Buffalo.

The paper before us appeared in the last number of the American Journal, and was undoubtedly perused with interest by those of our readers who subscribe to that stately periodical. The prognosis of fractures and dislocations has of late years occupied the attention of the author, who, with true philanthropic zeal, has made an elaborate report on this subject to the American Medical Association. This paper treats of the subject of compound dislocations briefly and with the author's well known clearness and decision, considering the subject especially in relation to the treatment by resection and reduction. The great objection to reduction with resection in opposition to simple reduction, is the shortening of the limb; but in the report to the American Med. Association, the author has shown that shortening of a limb to the extent of one-half or three-quarters of an inch, often fails to produce a halt in the gait. A universal appreciation of this fact would, we conceive, make the practice of reduction with resection, much more common than it is at present. F.

